# An Informal Internet Survey on the Current State of Consciousness Science

**DOI:** 10.3389/fpsyg.2018.02134

**Published:** 2018-11-05

**Authors:** Matthias Michel, Stephen M. Fleming, Hakwan Lau, Alan L. F. Lee, Susana Martinez-Conde, Richard E. Passingham, Megan A. K. Peters, Dobromir Rahnev, Claire Sergent, Kayuet Liu

**Affiliations:** ^1^Department of Philosophy, Sorbonne Université, Paris, France; ^2^Wellcome Centre for Human Neuroimaging, University College London, London, United Kingdom; ^3^Department of Psychology, University of California, Los Angeles, Los Angeles, CA, United States; ^4^Brain Research Institute, University of California, Los Angeles, Los Angeles, CA, United States; ^5^Department of Psychology, Hong State Key Laboratory of Brain and Cognitive Sciences, The University of Hong Kong, Pokfulam, Hong Kong; ^6^Department of Applied Psychology, Lingnan University, Tuen Mun, Hong Kong; ^7^SUNY Downstate Medical Center, State University of New York, New York, NY, United States; ^8^Department of Experimental Psychology, University of Oxford, Oxford, United Kingdom; ^9^Department of Bioengineering, University of California, Riverside, Riverside, CA, United States; ^10^School of Psychology, Georgia Institute of Technology, Atlanta, GA, United States; ^11^Laboratoire Psychologie de la Perception, Université Paris Descartes, CNRS, Paris, France; ^12^Department of Sociology, University of California, Los Angeles, Los Angeles, CA, United States

**Keywords:** consciousness, consciousness science, survey, meta-science, consciousness research

## Abstract

The scientific study of consciousness emerged as an organized field of research only a few decades ago. As empirical results have begun to enhance our understanding of consciousness, it is important to find out whether other factors, such as funding for consciousness research and status of consciousness scientists, provide a suitable environment for the field to grow and develop sustainably. We conducted an online survey on people’s views regarding various aspects of the scientific study of consciousness as a field of research. 249 participants completed the survey, among which 80% were in academia, and around 40% were experts in consciousness research. Topics covered include the progress made by the field, funding for consciousness research, job opportunities for consciousness researchers, and the scientific rigor of the work done by researchers in the field. The majority of respondents (78%) indicated that scientific research on consciousness has been making progress. However, most participants perceived obtaining funding and getting a job in the field of consciousness research as more difficult than in other subfields of neuroscience. Overall, work done in consciousness research was perceived to be less rigorous than other neuroscience subfields, but this perceived lack of rigor was not related to the perceived difficulty in finding jobs and obtaining funding. Lastly, we found that, overall, the global workspace theory was perceived to be the most promising (around 28%), while most non-expert researchers (around 22% of non-experts) found the integrated information theory (IIT) most promising. We believe the survey results provide an interesting picture of current opinions from scientists and researchers about the progresses made and the challenges faced by consciousness research as an independent field. They will inspire collective reflection on the future directions regarding funding and job opportunities for the field.

## Introduction

At the beginning of the 20th century, behaviorists wanted to constitute psychology as a scientific discipline by getting rid of mentalistic notions such as attention, memory, volition, mental imagery, and consciousness, which they regarded as unscientific (Watson, 1913; Skinner, 1953). This proved to be a mistake. With the rise of cognitive science, a wide variety of mental phenomena were progressively reintroduced within the scope of scientific investigation. However, while perceptual mechanisms were extensively studied, little effort was made to understand which mechanisms bring about consciousness of the contents of perception. The scientific study of consciousness became an organized field of research less than 30 years ago. Since then, a large number of empirical findings increased our understanding of consciousness (Block et al., 2014). Scientific progress, however, does not only consist of the advancement of knowledge (Kitcher, 1995). Increased funding for consciousness research and the rising status of scientists engaging in the field are also fundamental indicators of scientific progress as well as *sine qua non* conditions for such progress, and should not be overlooked. Here, we present the results of a survey designed to investigate the current state of the neuroscience of consciousness in regards to these latter aspects.

## Materials and Methods

Two hundred and fourty-nine subjects replied to our informal online survey. Participants were notified that they would not be compensated for their participation in the survey, and could stop taking the survey at any time. They were also informed that the purpose of the survey was to investigate their attitudes about current research on consciousness. Survey procedures were approved by the UCLA Institutional Review Board. Subjects completed the survey through the online software SurveyMonkey, by using their phone or computer. The survey was advertised through social media (e.g., Facebook, Twitter). As such, the survey did not involve random sampling or counterbalancing of question order, and it is also difficult to assess response rate; we will address these limitations further in Section “Discussion.” The survey was conducted between January 29, 2018 and February 14, 2018.

We asked 23 questions to the participants. We collected demographic data on the following: age, region, gender, year of highest degree, current position, number of publications, number of publications related to consciousness, number of publications on consciousness in public media, primary domain of expertise, conferences on consciousness attended (ASSC or Tucson), total amount of grants received for studying consciousness, overall amount of grants received.

Other questions concerned the possibility of a complete biological explanation of consciousness, progress in the understanding of consciousness thanks to neuroscience and psychology, improvement in the field of consciousness science, theories of consciousness perceived as most promising, experimental rigor of the work on consciousness compared to other subfields in neuroscience, difficulty of successfully competing for funding while studying consciousness compared to other neuroscience subfields, difficulty of the job market in consciousness science compared to other neuroscience subfields, preference for public or private funding of the work on consciousness, accuracy of popular media articles on consciousness, and perceived benefits of popular media article on consciousness.

For a full list of details of questions asked, please see Supplementary Data.

## Results

Of the 249 respondents, as many as 59% were active scholars (with >5 publications), and 29% were either tenure track or tenured academics. Overall, 80% of our participants were in academia. We achieved a good balance between experts in the field (43%, defined both as having attended ASSC—a conference organized by a professional society—or having published >5 papers on the topic), versus non-experts. 30% of participants were neuroscientists, 4% computer scientists, 20% philosophers, 37% psychologists, and 9% were working in other areas (humanities or natural/engineer sciences). 31% of the participants were primarily based in North America and 54% were based in Europe. On average, participants were 37 years old. 30% of participants were female, and 65% male (with 1% “Other” and 4% “Refuse to disclose”). See Supplementary Data for details.

### Progress

A great majority of participants (78%) agreed that progress has been made in the scientific study of consciousness. This result appears to hold regardless of whether one studies consciousness or not, as we found no dependence between expertise and perceived progress in the field [chi-squared tests, *p* = 0.32].

### Funding

**FIGURE 1:**
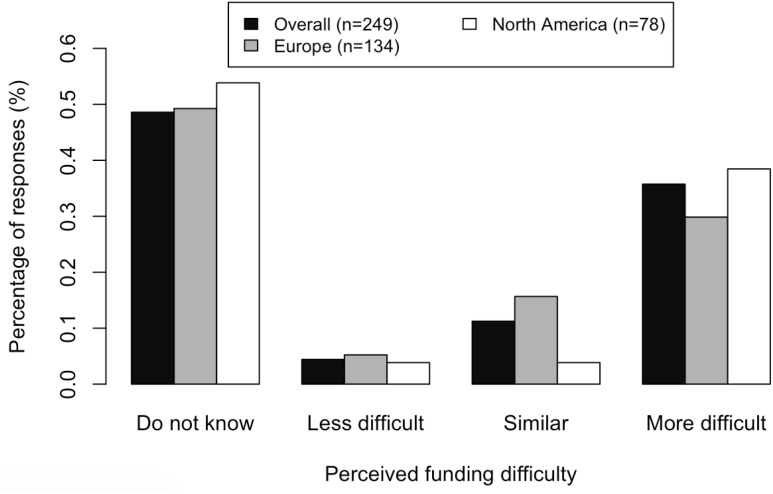
Responses to the question “*Compared to some other subfields in neuroscience, how difficult is it to successfully compete for funding for doing empirical work on consciousness?*” by primary region in which the respondents are based.

Many participants (36%) judged it more difficult to obtain funding for consciousness research than for other subfields of neuroscience they were familiar with (Figure 1). This was especially true for participants based primarily in North America, compared to those based primarily in Europe [Wilcoxon rank-sum test: *p* = 0.02]. We found no significant difference between experts and non-experts on the perceived difficulty of obtaining funding for studying consciousness [chi-squared test; *p* = 0.67].

### Jobs

**FIGURE 2:**
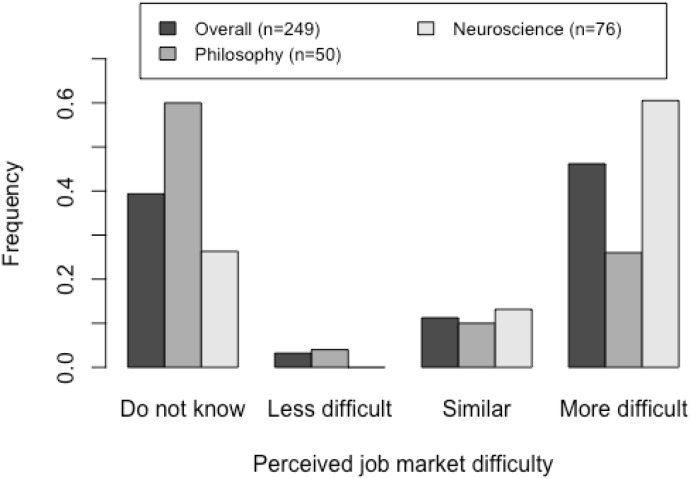
Responses to the question “*Compared to some other subfields in neuroscience, how difficult do you think it is for students and postdocs working primarily on consciousness to compete for faculty/independent principal investigator positions?*” by area of expertise of the respondent.

About half of participants reported that they believe it is more difficult to find a job in academia as a consciousness researcher than in other neuroscience subfields. Neuroscientists were more likely than those in other fields to respond that finding a job in consciousness science is more difficult compared to other subfields in neuroscience (Figure 2). We found no significant difference between experts and non-experts on perceived job-market difficulty [chi-squared test; *p* = 0.38].

### Rigor

More participants considered the work done in the field less rigorous than in other fields (34%), rather than the other way round (8%). This was true regardless of whether respondents worked on consciousness or not [chi-squared test; *p* = 0.65], which indicates that this is not just an outgroup bias; even experts within the field perceive lack of rigor as a problem.

This perceived lack of rigor does not explain the lack of funding and jobs, however. The field of consciousness was seen as similarly or more rigorous than fields such as social neuroscience (33% reported consciousness science as “more rigorous” than social neuroscience, versus only 10% who reported it as “less rigorous”), again both by experts as well as non-experts. Nonetheless, respondents reported that funding and jobs are found with greater difficulty for consciousness science than for social neuroscience (31% considered it more difficult to obtain funding for studying consciousness than social neuroscience, versus 6% who found it less difficult; 41% perceived the job market as more difficult for those who study consciousness compared to social neuroscience, versus 3% who found it less difficult).

### Role of Private Funding and Media

A large proportion of participants agreed that, for the healthy development of the field, public funding should be preferred over private funding (47 vs. 6%). This may be related to the concern that private funders might be misinformed by the depiction of the neuroscience of consciousness in popular media. Indeed, 44% of participants considered the representation of the neuroscience of consciousness in popular media to be less accurate than the representation of other scientific topics, whereas only 5% found it more accurate. The below section contains a possibly relevant and interesting example of media influence.

### Theories Perceived as Most Promising

We asked participants to rate which consciousness theory they perceived to be most promising. Overall, the global workspace theory (Dehaene and Changeux, 2011; Dehaene et al., 2014) was seen as the most promising theory of consciousness (28%). On the other hand, among non-experts only, IIT [Integrated Information Theory; (Oizumi et al., 2014; Tononi et al., 2016)] was considered to be the most promising theory (22% of non-experts); this was true whether expertise was defined as having published more than five articles on consciousness (chi-squared test, *p* < 0.05), or attending ASSC at least once (chi-squared test, *p* < 0.05).

We suspect that one explanation for this may be that IIT has been promoted heavily in popular media, with such claims as “[IIT is the] only really promising fundamental theory of consciousness,” appearing in influential media outlets^1^. Non-experts may be particularly influenced by these claims. We also cannot rule out other possibilities, however, such that non-experts may be particularly impressed by mathematical complexity (a feature of IIT relative to other theories presented in our survey).

For further details about the responses to each of the questions asked, please see Supplementary Data.

## Discussion

The field of consciousness research is perceived as making progress. However, the field also faces significant challenges that could overthrow the progress accomplished so far.

We emphasize that this survey was informal and that our results are subject to significant limitations. For example, the sample size of 249 participants may be considered small. However, we note that the typical attendance to ASSC is under 500 people. Hence, relative to the size of the field, including over a 100 experts plus a group of non-experts of similar size is not uninformative.

**FIGURE 3:**
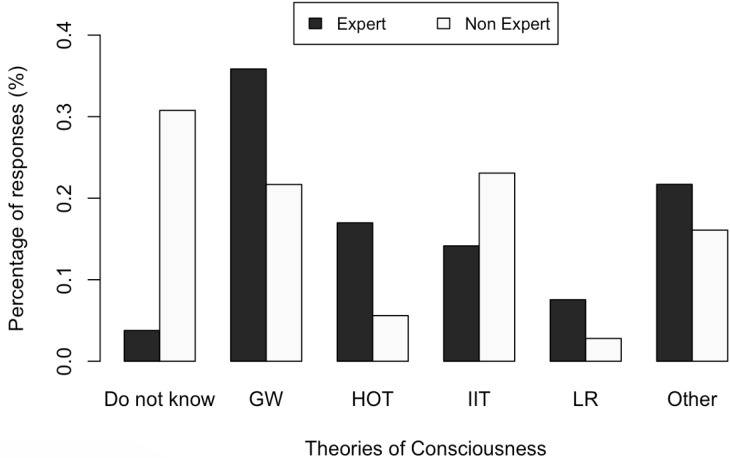
*Comparison between answers from experts and non-experts to the question: “To the extent that you have read/heard about them, which of the following theories seem most promising to you?”* GW, Global Workspace Theory; HOT, Higher-Order Theory; IIT, Integrated Information Theory; LR, Local Recurrence Theory.

Moreover, since the survey was advertised through social media, the opinions of our participants might reflect some degree of selection bias. It is possible that respondents selected through social media could be more sympathetic to the overall project of a neuroscience of consciousness than the overall scientific population. However, as can be seen in the data, e.g., Figure 3, a fairly broad group of experts were included in the survey (*n* = 106), in addition to a good number of non-experts (*n* = 143). The comparisons made in the report highlight that many of the perceived notions about the field are common to both groups.

It could be argued that in our sampling, participants may be biased in favor of consciousness science, because those who answered the survey were probably interested in consciousness science in the first place. But it is important to note that most participants considered that the science of consciousness was *less* rigorous than work done in other disciplines. While this does not mean that respondents were uninterested in consciousness, it does suggests that they were not unconditional promoters of consciousness research. Importantly, many of the interesting results concern comparisons between experts and non-experts within the cohort. Biases that apply to the entire cohort should affect overall patterns, but not the comparisons between subgroups (where biases should be largely subtracted out). Finally, as we noted above, the sample size is not small relative to the size of the field.

One could also argue that our definitions of the categories of “experts” and “non-experts” do not correctly represent expertise on consciousness science. However, non-experts did appear to have a lower degree of knowledge of the field than experts, as they selected “don’t know” answers more often on all questions (as reflected, for example, in Figure 3). Moreover, our results did not significantly vary depending on how we defined expertise (either as publishing more than five articles on consciousness, or as attending to the ASSC at least once).

Finally, when questioning preferred theories, we did not represent illusionism, quantum theories of consciousness, or the view that no satisfying theory of consciousness can be developed. As such, these theories were only represented indirectly, as belonging to the category “other.” This might have biased results in favor of the various theories explicitly represented. Although we note that this could be a problem, it is unlikely that any of the four theories that we presented should have particularly benefited from this limitation. Hence, the overall distribution of preferences between theories would probably have been preserved even if all theories of consciousness were represented.

Moreover, even if the absence of some theories such as illusionism or quantum theories of consciousness might have biased the choice in favor of represented theories, this bias probably did not affect the overall pattern of responses as a function of expertise.

Overall, despite these caveats, this survey provides an interesting picture of the current opinion of a group of scientists on the progresses and challenges in the field of consciousness, and we hope that it might spark a collective reflection on the future of funding policies in the domain.

## Ethics Statement

This study was carried out in accordance with the recommendations from of the UCLA Office of the Human Research Protection Program. The protocol was approved by the UCLA Institutional Review Board.

## Author Contributions

MM and HL contributed to the conception and design of the study. MM organized the database, performed the statistical analysis, and wrote the first draft of the manuscript. All authors contributed to manuscript revision, read, and approved the submitted version.

## Conflict of Interest Statement

The authors declare that the research was conducted in the absence of any commercial or financial relationships that could be construed as a potential conflict of interest. The reviewer DR declared a past co-authorship with one of the authors HL to the handling Editor.

## Supplementary Material

The Supplementary Material for this article can be found online at: https://www.frontiersin.org/articles/10.3389/fpsyg.2018.02134/full#supplementary-material

## Footnotes

1 See, e.g., http://www.nytimes.com/2010/09/21/science/21consciousness.html, https://qz.com/709969/2300-years-later-platos-theory-of-consciousness-is-being-backed-up-by-neuroscience/, https://www.newyorker.com/tech/elements/how-much-consciousness-does-an-iphone-have, or https://www.huffingtonpost.com/bobby-azarian/post_10079_b_8160914.html.
