# Supplementary material for: An Informal Internet Survey on the Current State of Consciousness Science
Source: Front Psychol. 2018 Nov 5;9:2134. doi: 10.3389/fpsyg.2018.02134 (PMC6230957; doi:10.3389/fpsyg.2018.02134)
Supplement: Supplementary file 1 [file Data_Sheet_1.PDF]

Q1 Do you think that there could ever be a complete biological explanation of consciousness ?

Answered: 249 Skipped: 0

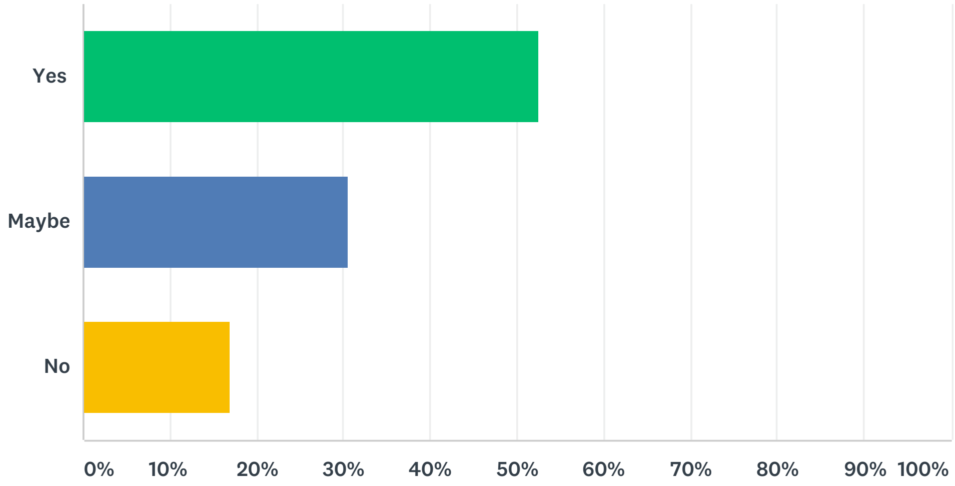

ANSWER CHOICES	RESPONSES	
Yes	52.61%	131
Maybe	30.52%	76
No	16.87%	42
TOTAL		249

Q2 Do you think that there has been meaningful progress so far on the study of consciousness thanks to neuroscience and psychology?

Answered: 249 Skipped: 0

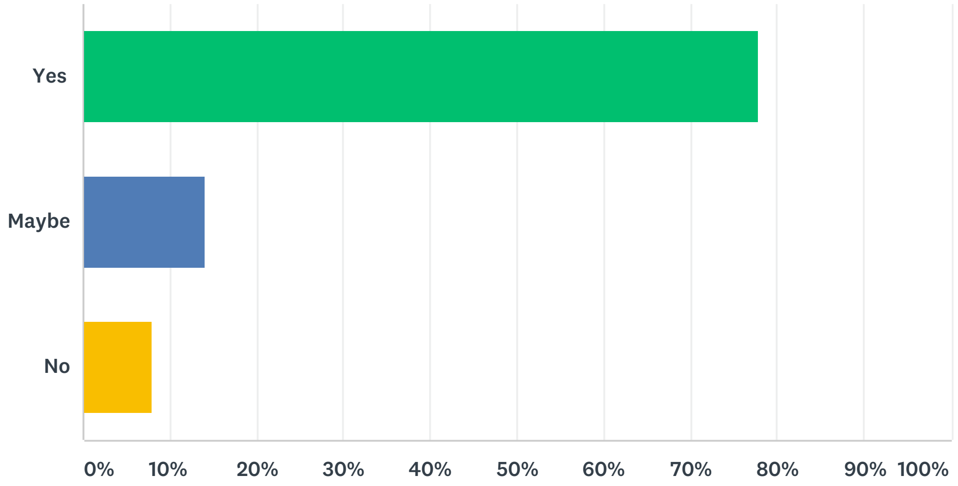

ANSWER CHOICES	RESPONSES	
Yes	77.91%	194
Maybe	14.06%	35
No	8.03%	20
TOTAL		249

Q3 Specifically, regarding the past 5 years, do you think the field of consciousness has improved (in terms of rigor, insights, and impact)?

Answered: 249 Skipped: 0

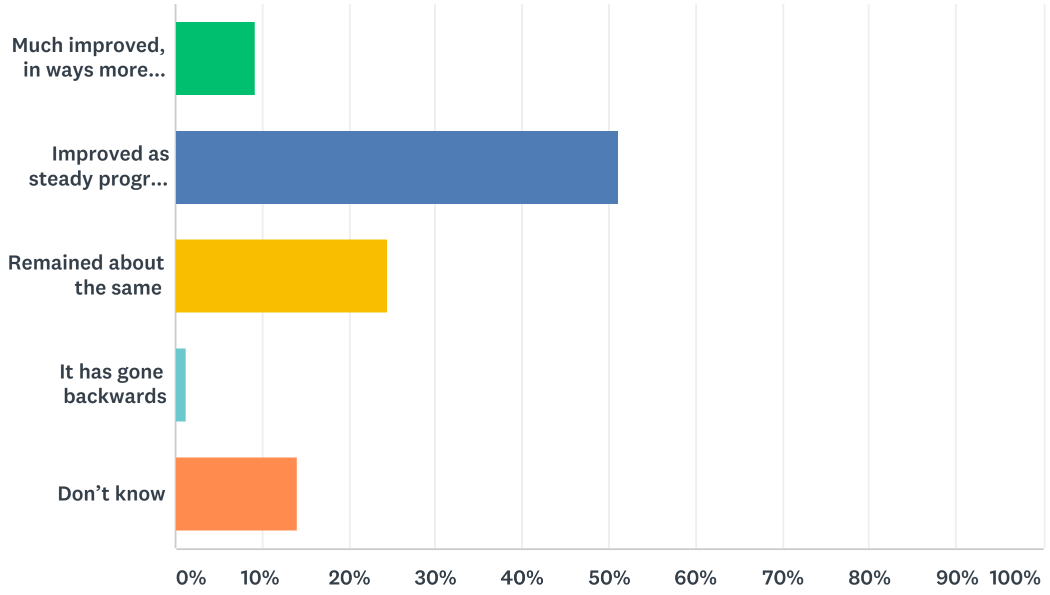

ANSWER CHOICES	RESPONSES	
Much improved, in ways more than expected from past trajectory	9.24%	23
Improved as steady progress has been made	51.00%	127
Remained about the same	24.50%	61
It has gone backwards	1.20%	3
Don’t know	14.06%	35
TOTAL		249

Q4 To the extent that you have read/heard about them, which of the following theories seem most promising to you?

Answered: 249   Skipped: 0

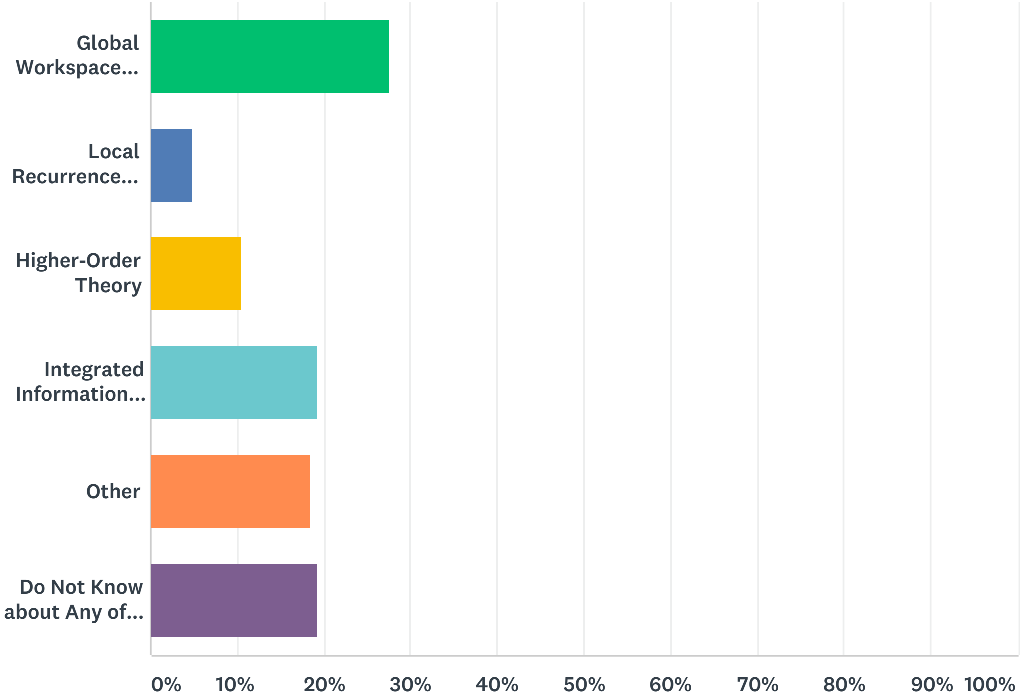

ANSWER CHOICES	RESPONSES	
Global Workspace Theory	27.71%	69
Local Recurrence Theory	4.82%	12
Higher-Order Theory	10.44%	26
Integrated Information Theory	19.28%	48
Other	18.47%	46
Do Not Know about Any of Them	19.28%	48
TOTAL		249

Q5 Have you ever attended these conferences on consciousness?

Answered: 249 Skipped: 0

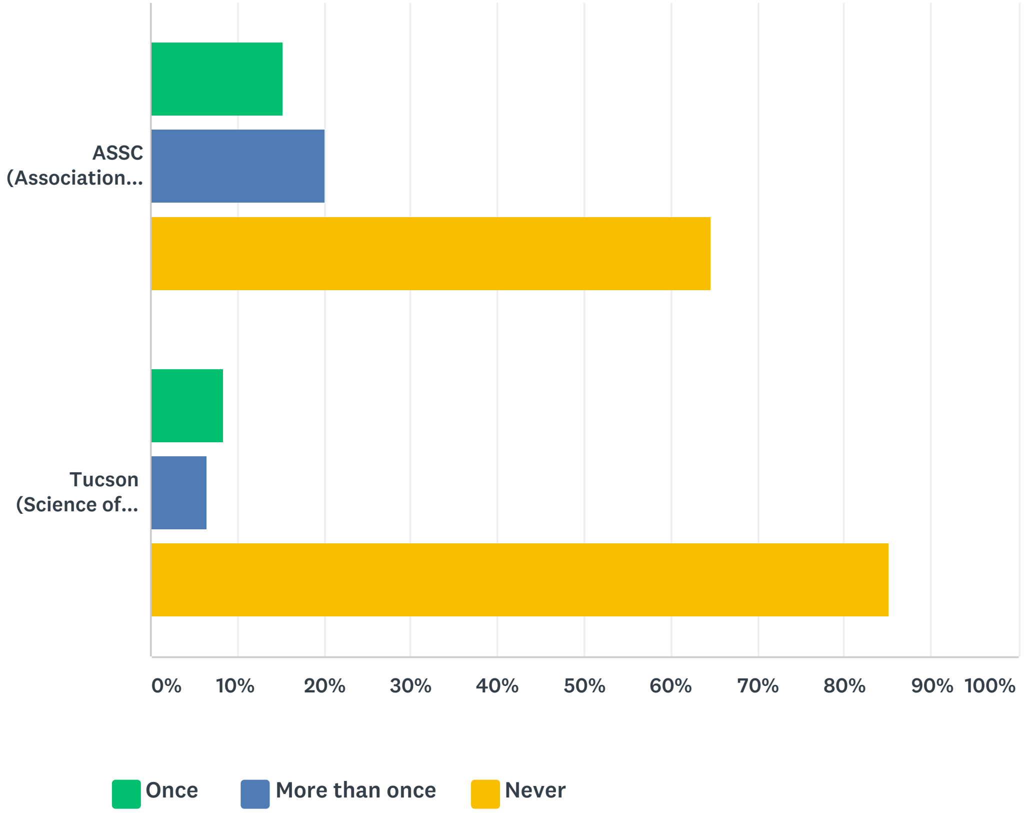

	ONCE	MORE THAN ONCE	NEVER	TOTAL
ASSC (Association for the Scientific Studies of Consciousness)	15.26%<br>38	20.08%<br>50	64.66%<br>161	249
Tucson (Science of Consciousness)	8.43%<br>21	6.43%<br>16	85.14%<br>212	249

Q6 Compared to some other subfields in neuroscience, how experimentally rigorous is the work on consciousness in general?

Answered: 249 Skipped: 0

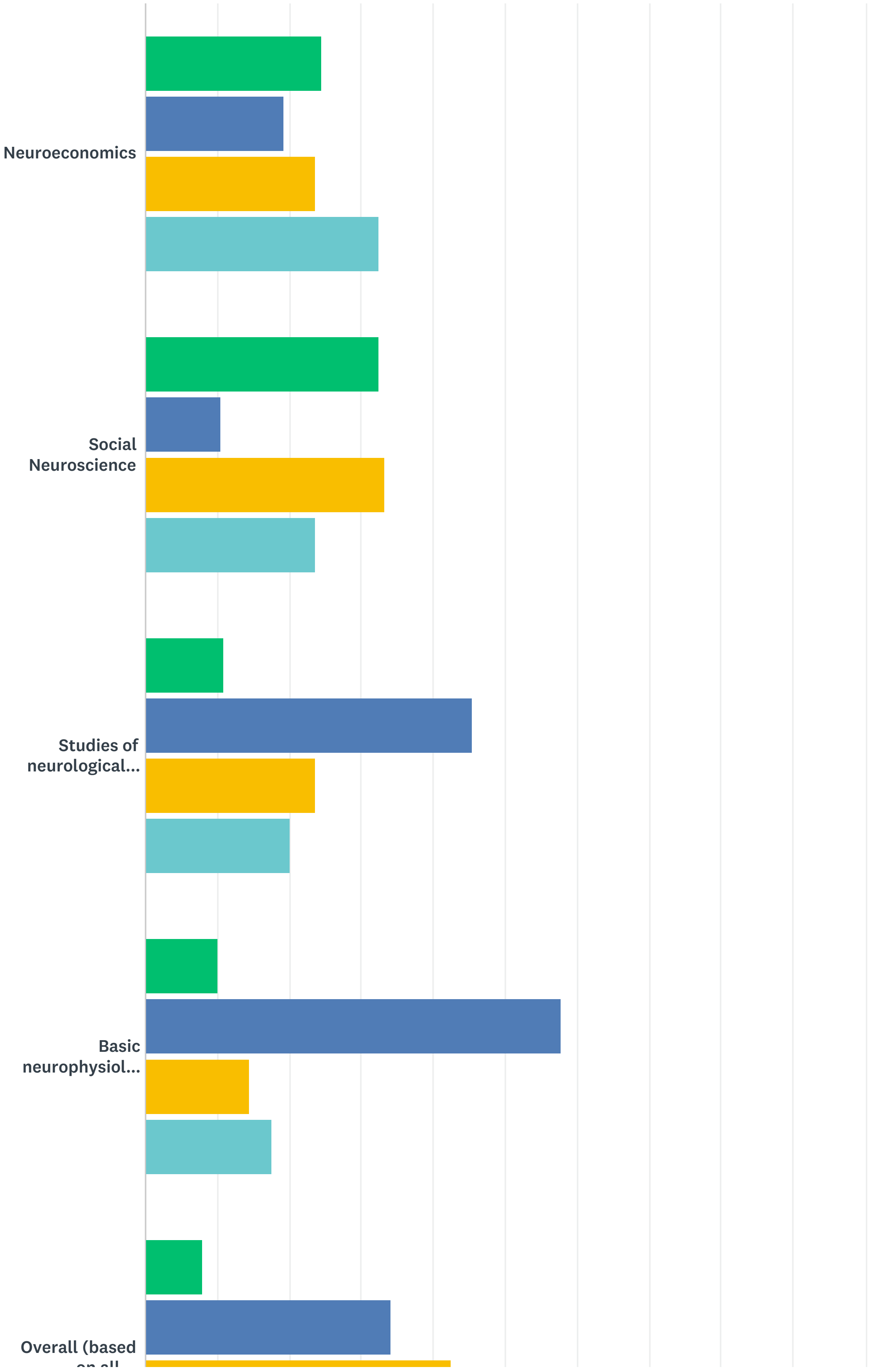

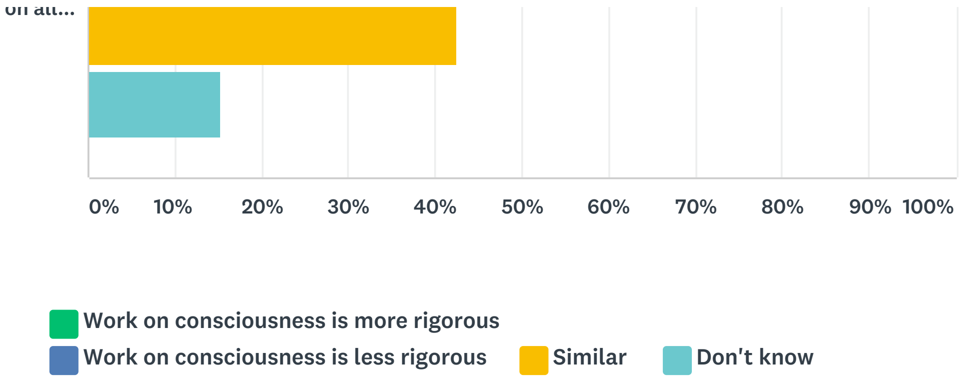

	WORK ON CONSCIOUSNESS IS MORE RIGOROUS	WORK ON CONSCIOUSNESS IS LESS RIGOROUS	SIMILAR	DON'T KNOW	TOTAL
Neuroeconomics	24.50%<br>61	19.28%<br>48	23.69%<br>59	32.53%<br>81	249
Social Neuroscience	32.53%<br>81	10.44%<br>26	33.33%<br>83	23.69%<br>59	249
Studies of neurological diseases (e.g. Parkinson's, Alzheimer's)	10.84%<br>27	45.38%<br>113	23.69%<br>59	20.08%<br>50	249
Basic neurophysiology in animal models (e.g. optogenetics in rodents, multi-unit recording in non-human primates)	10.04%<br>25	57.83%<br>144	14.46%<br>36	17.67%<br>44	249
Overall (based on all subfields you are familiar with, including but not limited to the ones above)	8.03%<br>20	34.14%<br>85	42.57%<br>106	15.26%<br>38	249

Q7 Compared to some other subfields in neuroscience, how difficult is it to successfully compete for funding for doing empirical work on consciousness?

Answered: 249 Skipped: 0

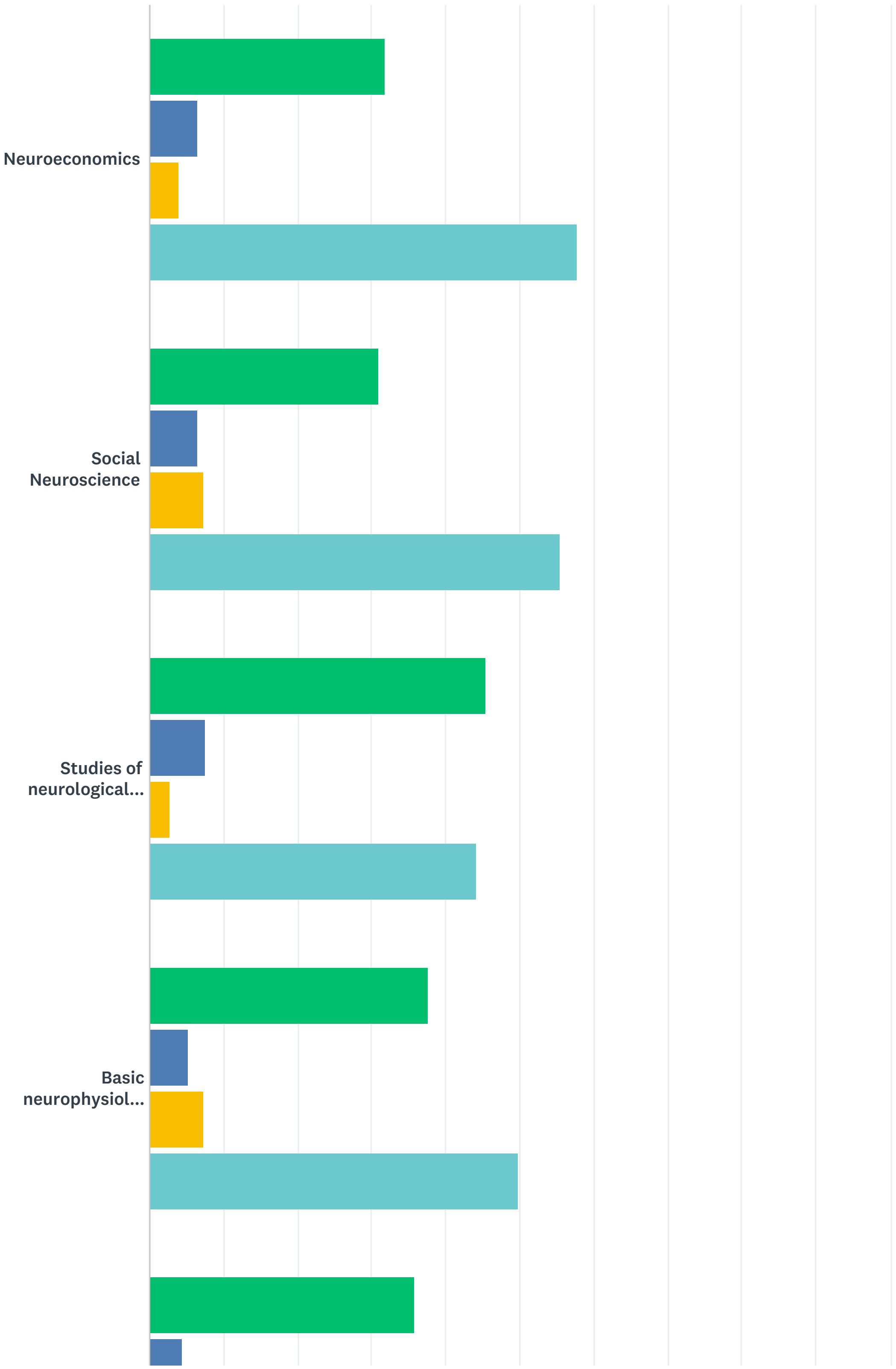

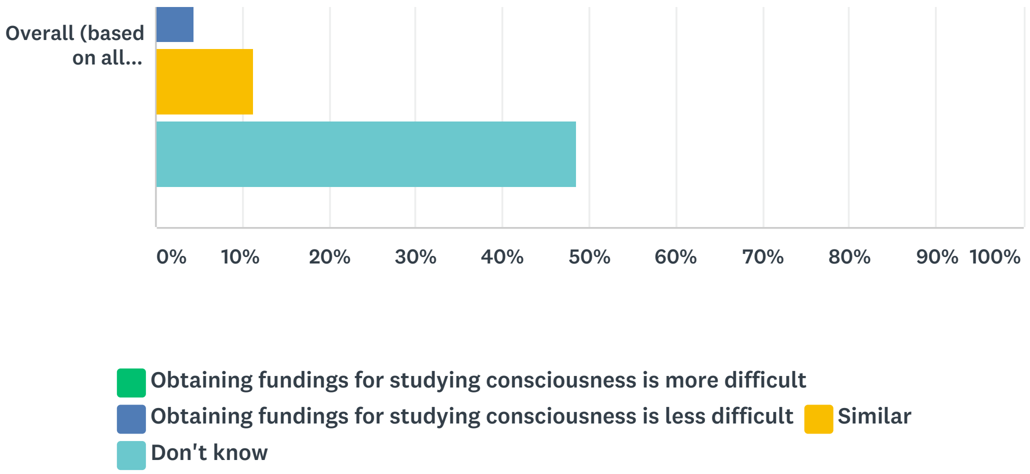

	OBTAINING FUNDINGS FOR STUDYING CONSCIOUSNESS IS MORE DIFFICULT	OBTAINING FUNDINGS FOR STUDYING CONSCIOUSNESS IS LESS DIFFICULT	SIMILAR	DON'T KNOW	TOTAL
Neuroeconomics	31.73%<br>79	6.43%<br>16	4.02%<br>10	57.83%<br>144	249
Social Neuroscience	30.92%<br>77	6.43%<br>16	7.23%<br>18	55.42%<br>138	249
Studies of neurological diseases (e.g. Parkinson's, Alzheimer's)	45.38%<br>113	7.63%<br>19	2.81%<br>7	44.18%<br>110	249
Basic neurophysiology in animal models (e.g. optogenetics in rodents, multi-unit recording in non-human primates)	37.75%<br>94	5.22%<br>13	7.23%<br>18	49.80%<br>124	249
Overall (based on all subfields you are familiar with, including but not limited to the ones above)	35.74%<br>89	4.42%<br>11	11.24%<br>28	48.59%<br>121	249

Q8 Compared to some other subfields in neuroscience, how difficult do you think it is for students and postdocs working primarily on consciousness to compete for faculty / independent principal investigator positions?

Answered: 249 Skipped: 0

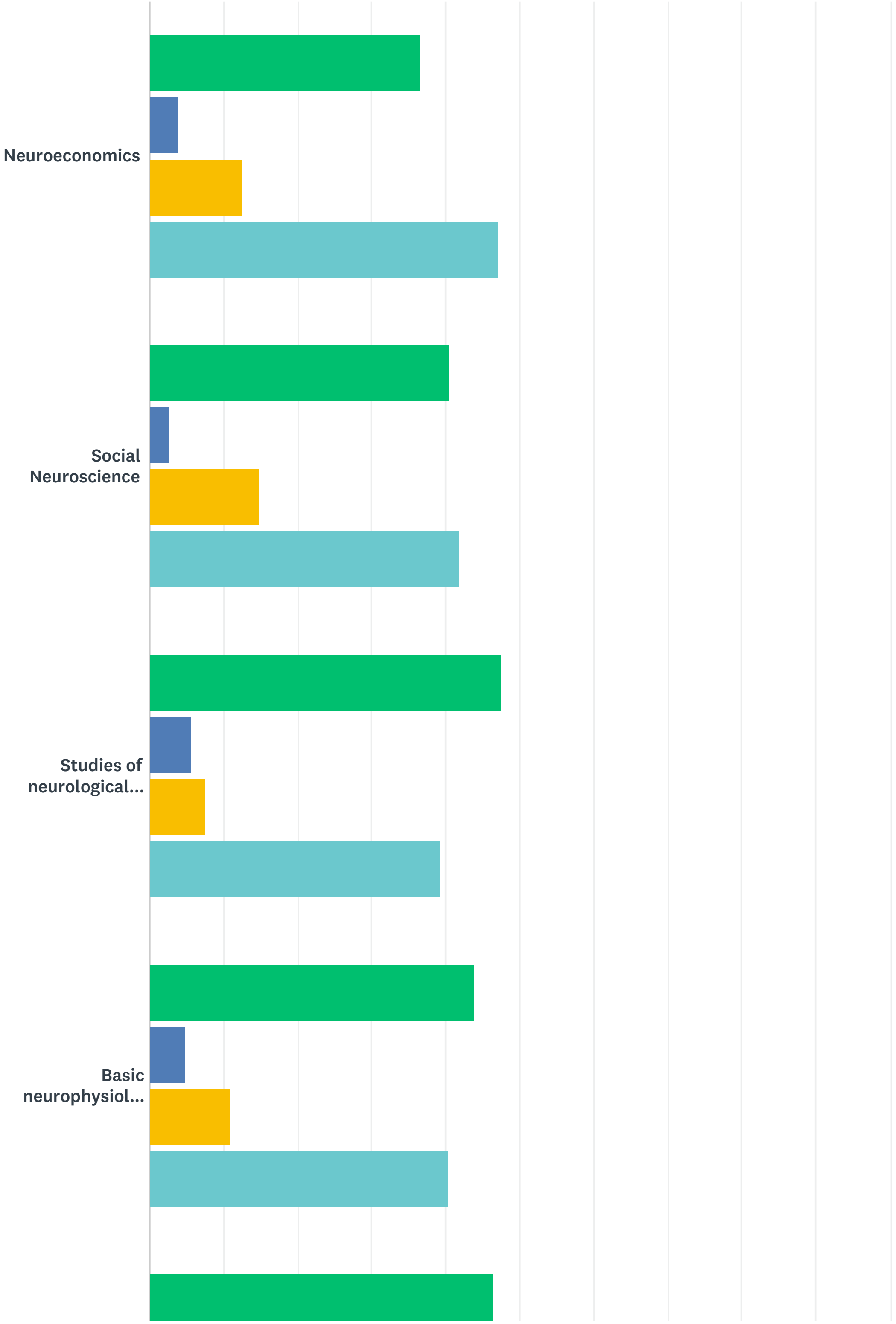

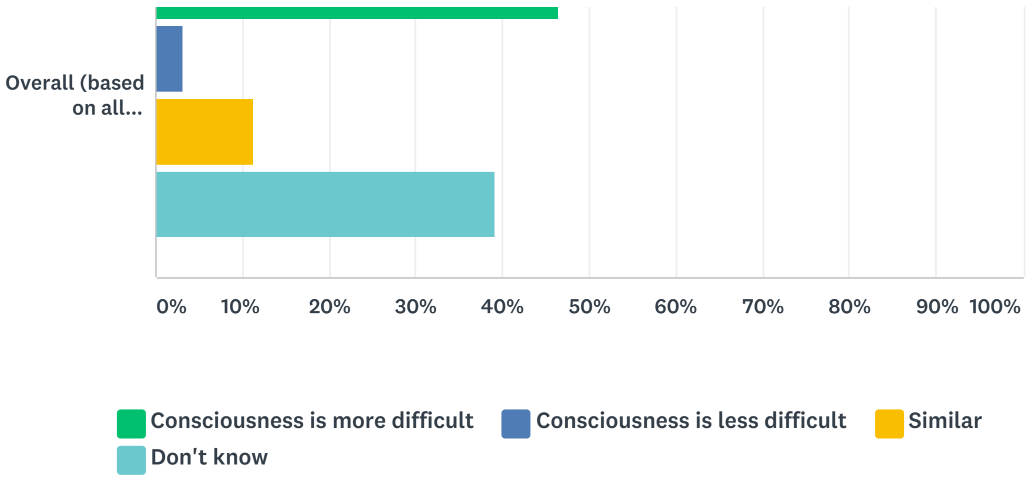

	CONSCIOUSNESS IS MORE DIFFICULT	CONSCIOUSNESS IS LESS DIFFICULT	SIMILAR	DON'T KNOW	TOTAL
Neuroeconomics	36.55% 91	4.02% 10	12.45% 31	46.99% 117	249
Social Neuroscience	40.56% 101	2.81% 7	14.86% 37	41.77% 104	249
Studies of neurological diseases (e.g. Parkinson’s, Alzheimer’s)	47.39% 118	5.62% 14	7.63% 19	39.36% 98	249
Basic neurophysiology in animal models (e.g. optogenetics in rodents, multi-unit recording in non-human primates)	43.95% 109	4.84% 12	10.89% 27	40.32% 100	248
Overall (based on all subfields you are familiar with, including but not limited to the ones above)	46.37% 115	3.23% 8	11.29% 28	39.11% 97	248

Q9 Do you think consciousness research would improve if the field relied less on private funding, and more on mainstream governmental support (e.g. NSF / NSF / ERC)

Answered: 249 Skipped: 0

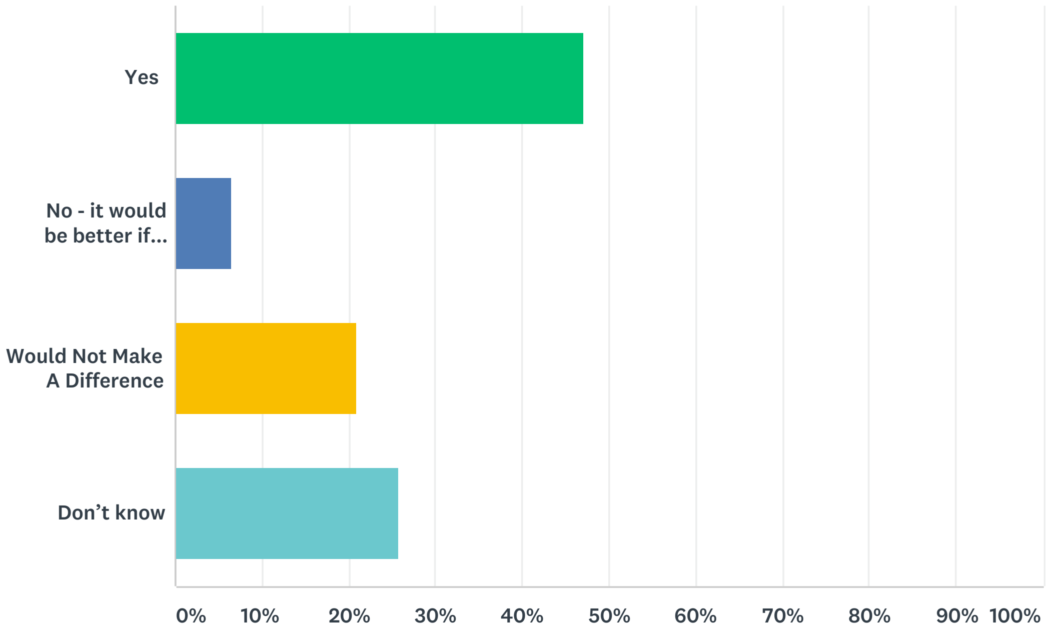

ANSWER CHOICES	RESPONSES	
Yes	46.99%	117
No - it would be better if it is driven more by private funding	6.43%	16
Would Not Make A Difference	20.88%	52
Don't know	25.70%	64
TOTAL		249

Q10 It is sometimes lamented that science journalism in the popular media does not always accurately reflect what we really know and don’t know based on actual research. Do you think this problem is worse for consciousness in particular?

Answered: 249 Skipped: 0

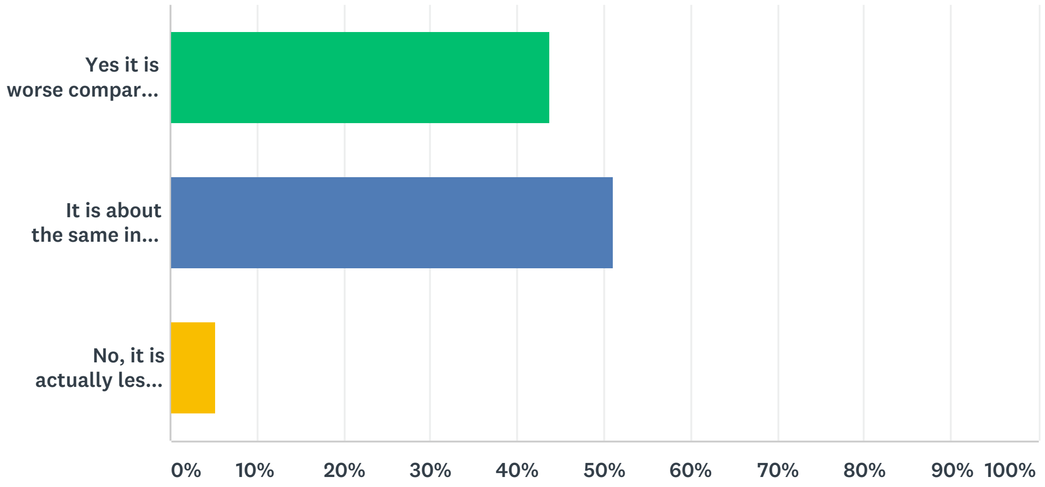

ANSWER CHOICES	RESPONSES	
Yes it is worse compared to other subfields in neuroscience / psychology	43.78%	109
It is about the same in other subfields in neuroscience / psychology	51.00%	127
No, it is actually less of a problem in consciousness compared to other subfields in neuroscience / psychology	5.22%	13
TOTAL		249

Q11 Overall, do you think consciousness research would benefit if it received more media attention?

Answered: 249 Skipped: 0

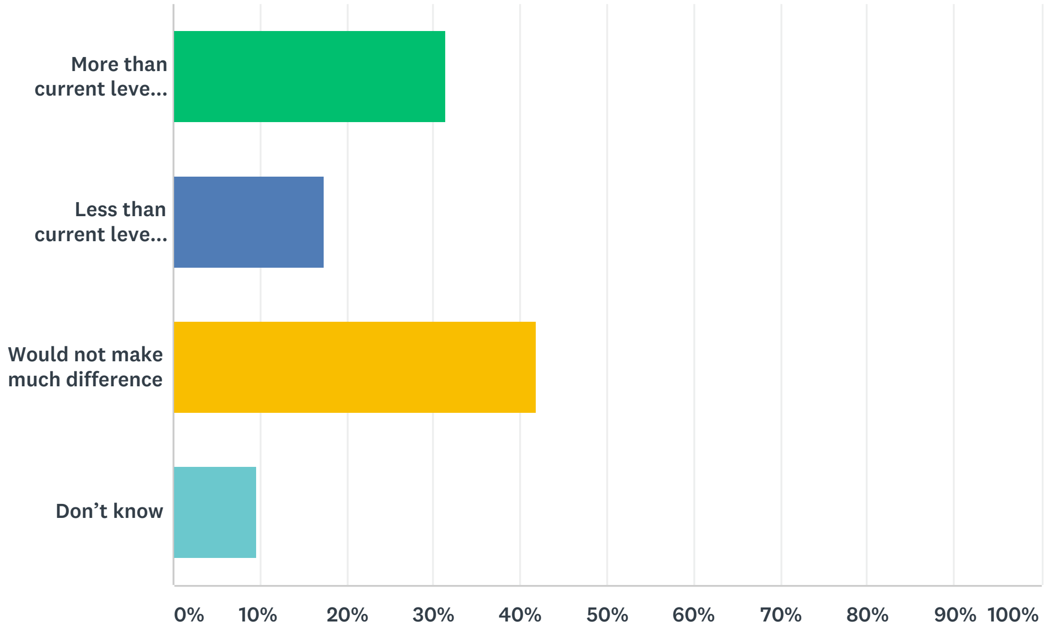

ANSWER CHOICES	RESPONSES	
More than current level would be better	31.33%	78
Less than current level would be better	17.27%	43
Would not make much difference	41.77%	104
Don’t know	9.64%	24
TOTAL		249

Q12 Assuming the total funding for biomedical sciences is fixed, and that certain subfields are considered by some to have high priority (e.g. clinical applications, development of basic methods), do you think consciousness research should receive more or less funding than the current level?

Answered: 249 Skipped: 0

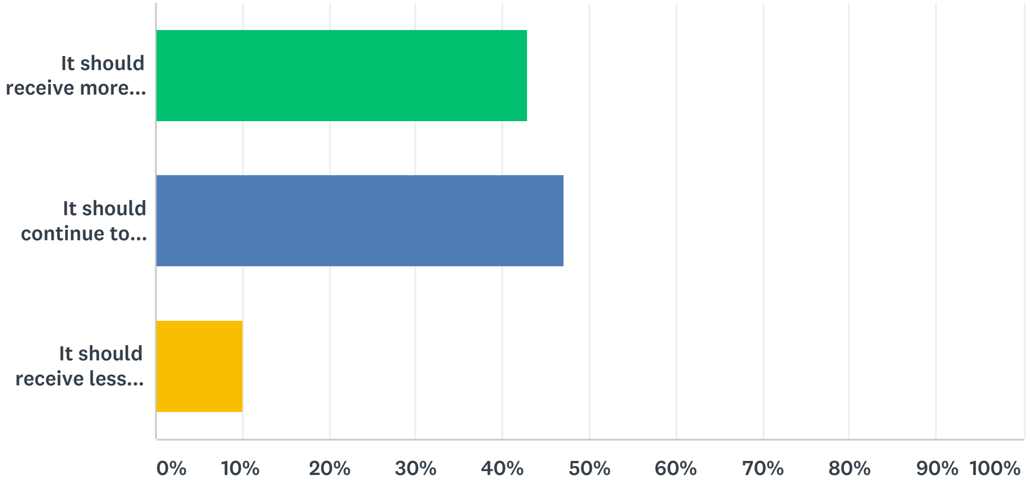

ANSWER CHOICES	RESPONSES	
It should receive more funding (meaning we would need to be taking funding away from other subfields)	42.97%	107
It should continue to receive its current level of funding	46.99%	117
It should receive less funding (so that we can give more funding to other subfields)	10.04%	25
TOTAL		249

Q13 Your primary domain of expertise

Answered: 249 Skipped: 0

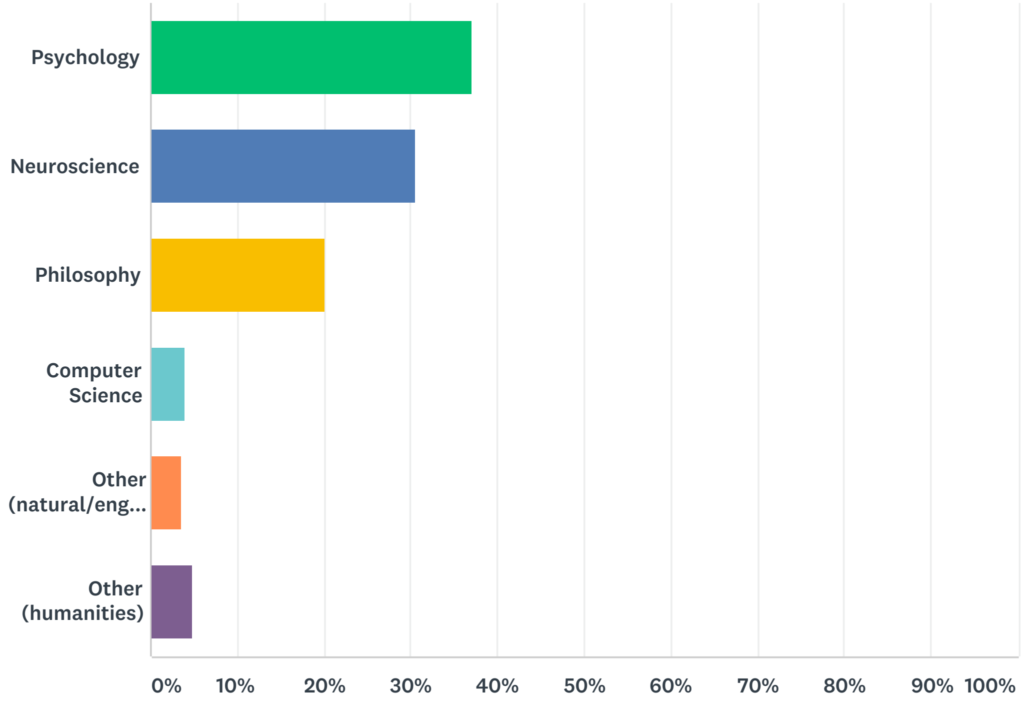

ANSWER CHOICES	RESPONSES	
Psychology	36.95%	92
Neuroscience	30.52%	76
Philosophy	20.08%	50
Computer Science	4.02%	10
Other (natural/engineer sciences)	3.61%	9
Other (humanities)	4.82%	12
TOTAL		249

Q14 Region you are currently primarily based

Answered: 249 Skipped: 0

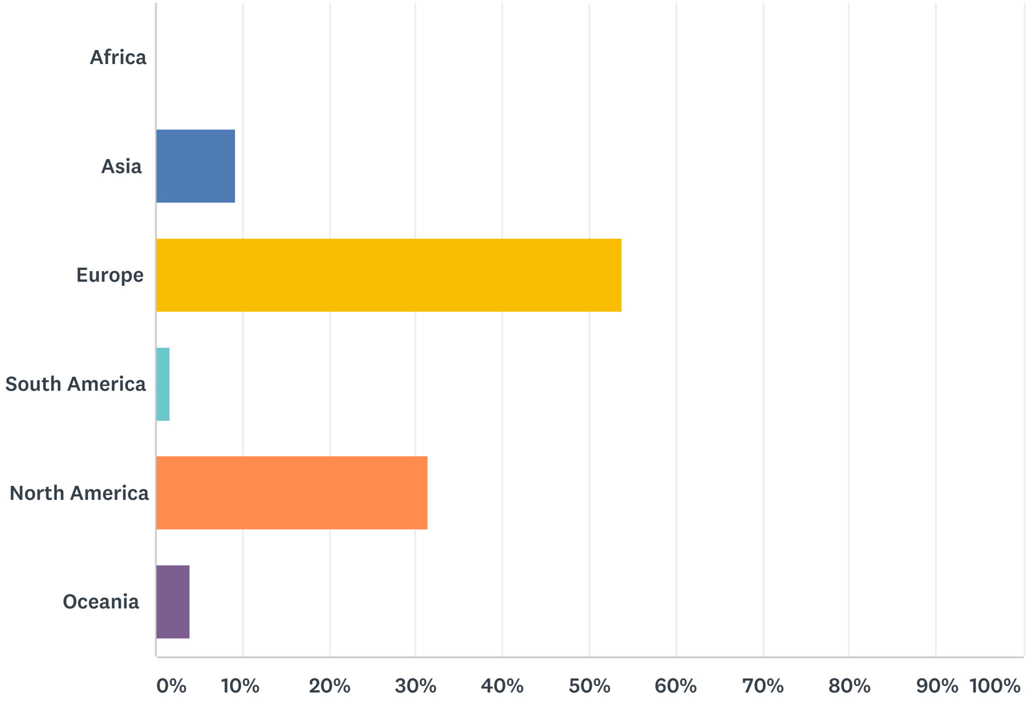

ANSWER CHOICES	RESPONSES	
Africa	0.00%	0
Asia	9.24%	23
Europe	53.82%	134
South America	1.61%	4
North America	31.33%	78
Oceania	4.02%	10
TOTAL		249

Q15 Gender

Answered: 249 Skipped: 0

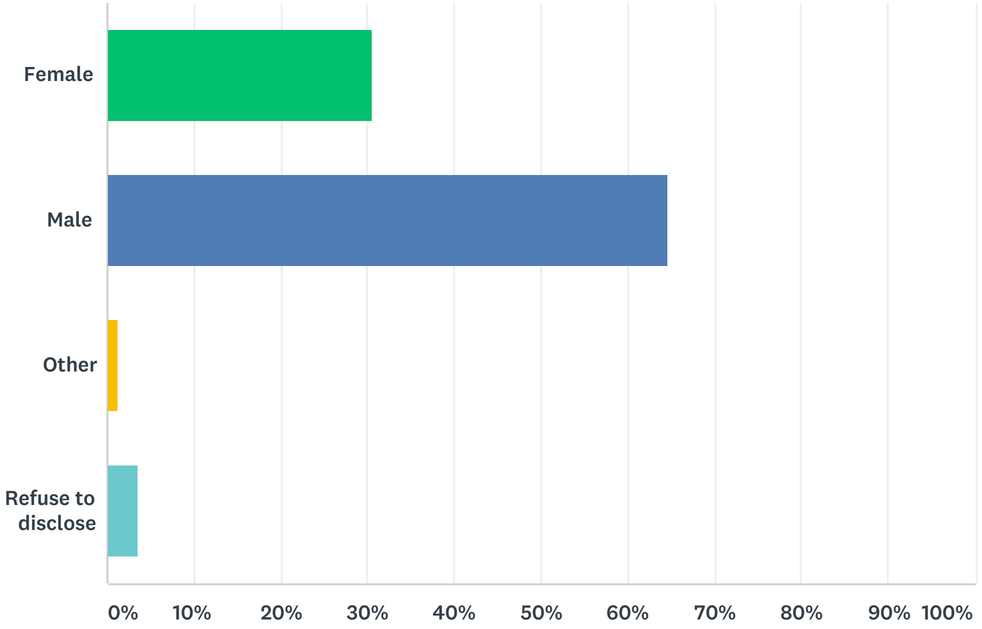

ANSWER CHOICES	RESPONSES	
Female	30.52%	76
Male	64.66%	161
Other	1.20%	3
Refuse to disclose	3.61%	9
TOTAL		249

Q16 Age

Answered: 249   Skipped: 0

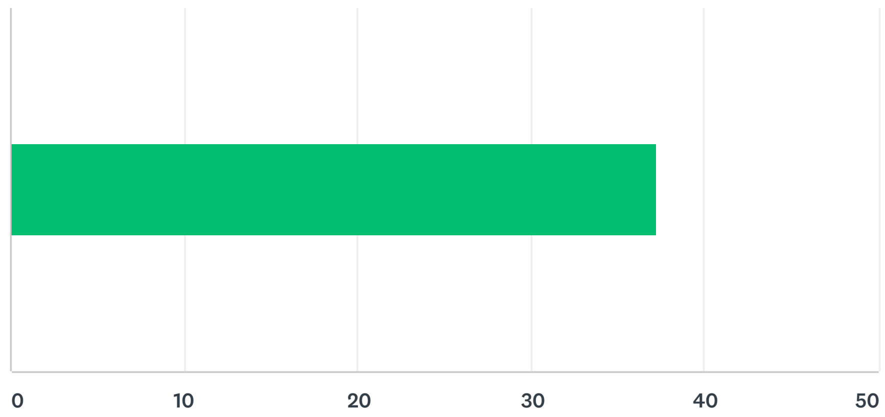

ANSWER CHOICES	AVERAGE NUMBER	TOTAL NUMBER	RESPONSES
	37	9,283	249
Total Respondents: 249			

Q17 Year highest degree earned

Answered: 249 Skipped: 0

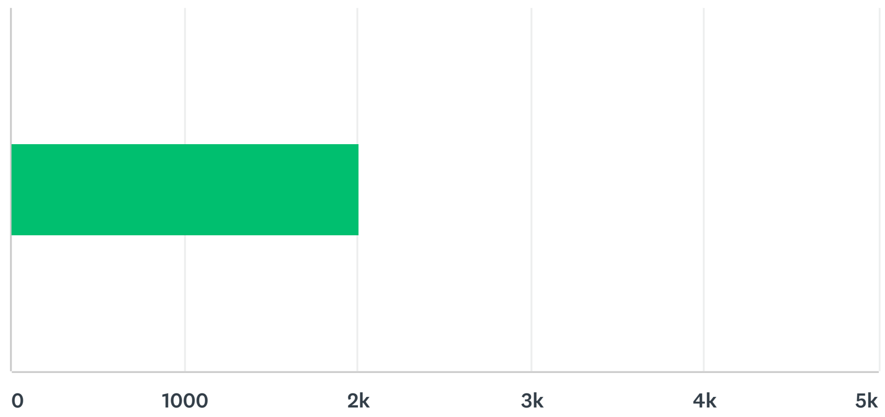

ANSWER CHOICES	AVERAGE NUMBER	TOTAL NUMBER	RESPONSES
	2,010	500,472	249
Total Respondents: 249			

Q18 Job status:

Answered: 249 Skipped: 0

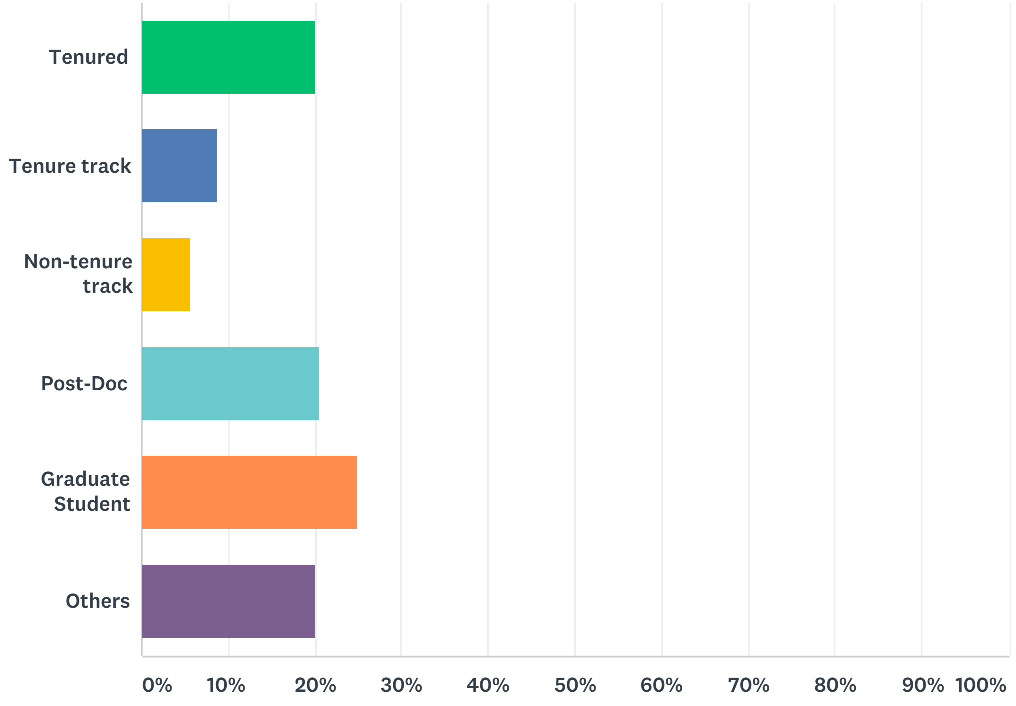

ANSWER CHOICES	RESPONSES	
Tenured	20.08%	50
Tenure track	8.84%	22
Non-tenure track	5.62%	14
Post-Doc	20.48%	51
Graduate Student	24.90%	62
Others	20.08%	50
TOTAL		249

Q19 Number of articles published

Answered: 249 Skipped: 0

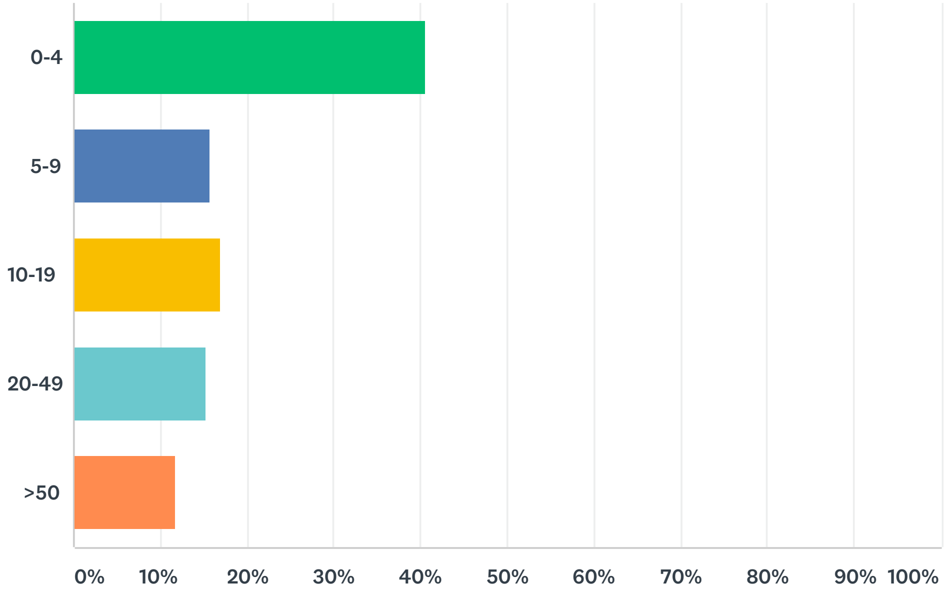

ANSWER CHOICES	RESPONSES	
0-4	40.56%	101
5-9	15.66%	39
10-19	16.87%	42
20-49	15.26%	38
>50	11.65%	29
TOTAL		249

Q20 Number of articles directly relevant to consciousness

Answered: 249 Skipped: 0

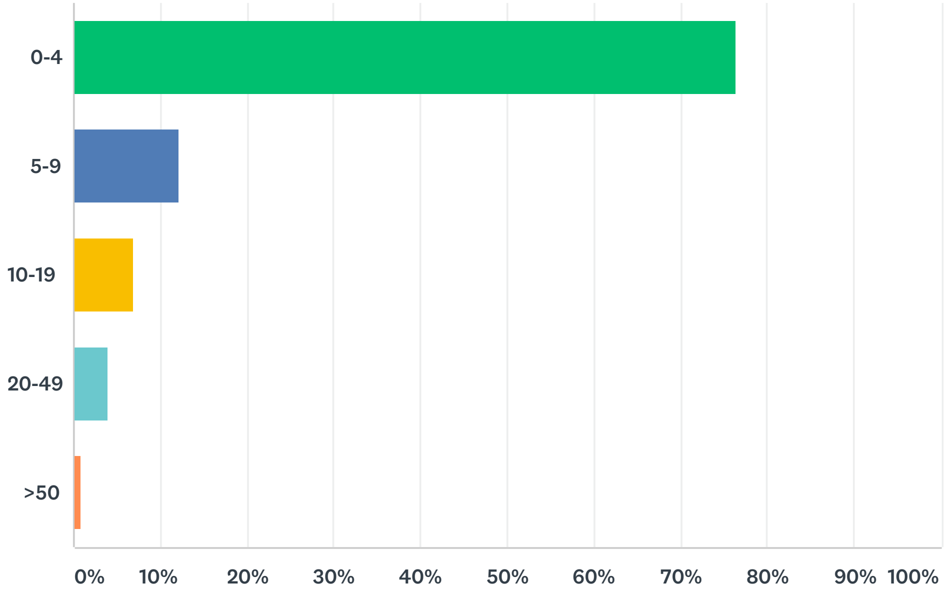

ANSWER CHOICES	RESPONSES	
0-4	76.31%	190
5-9	12.05%	30
10-19	6.83%	17
20-49	4.02%	10
>50	0.80%	2
TOTAL		249

Q21 Publications in public media on consciousness

Answered: 249 Skipped: 0

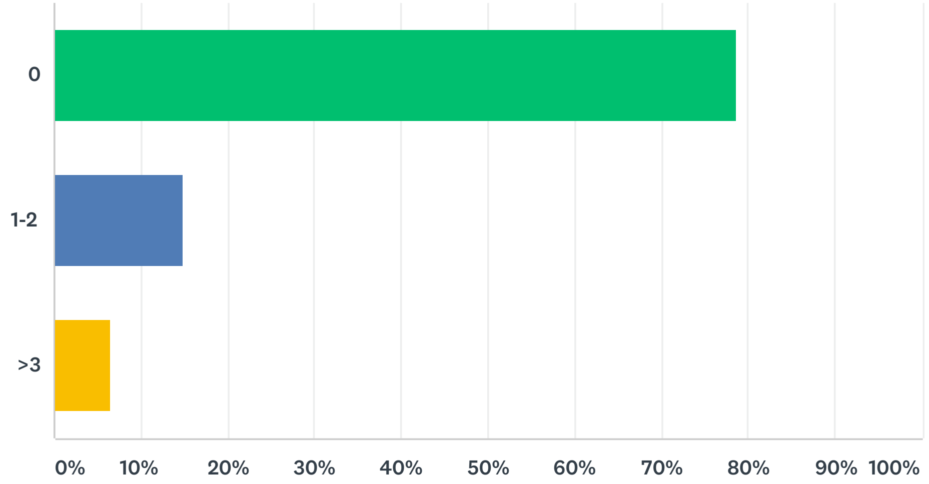

ANSWER CHOICES	RESPONSES	
0	78.71%	196
1-2	14.86%	37
>3	6.43%	16
TOTAL		249

Q22 Total amount of grants/fundings received for studying consciousness (direct costs):

Answered: 249 Skipped: 0

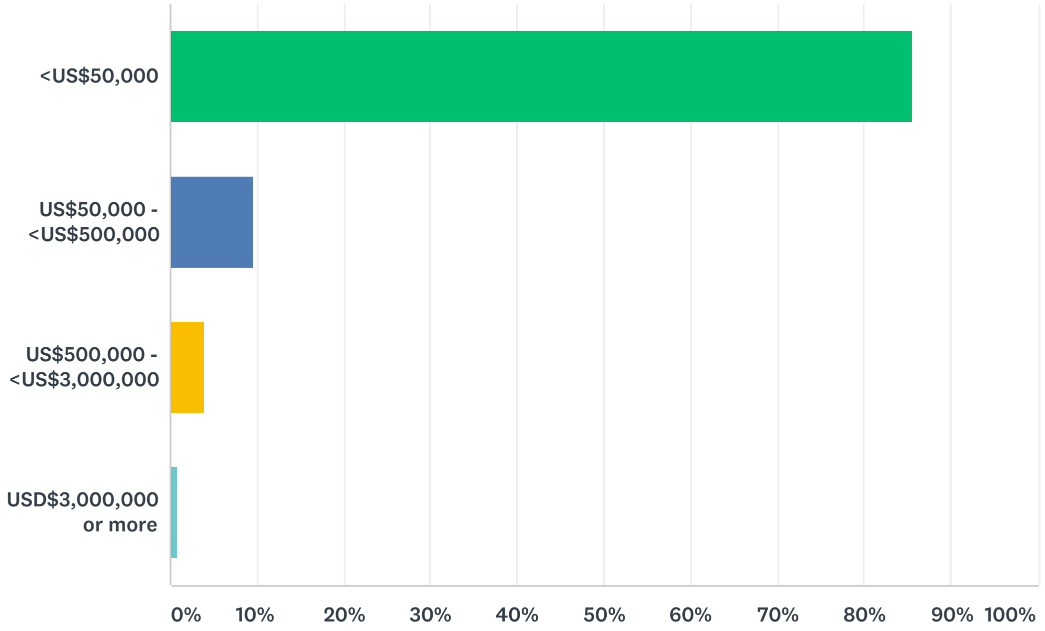

ANSWER CHOICES	RESPONSES	
<US$50,000	85.54%	213
US$50,000 - <US$500,000	9.64%	24
US$500,000 - <US$3,000,000	4.02%	10
USD$3,000,000 or more	0.80%	2
TOTAL		249

Q23 Total amount of grants/fundings received (direct costs):

Answered: 249 Skipped: 0

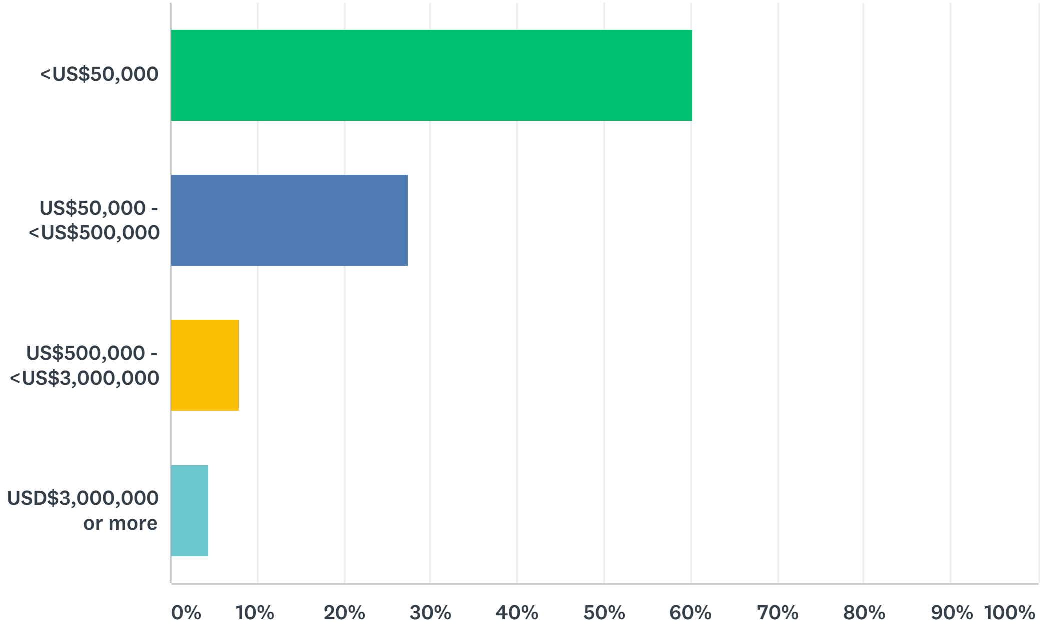

ANSWER CHOICES	RESPONSES	
<US$50,000	60.24%	150
US$50,000 - <US$500,000	27.31%	68
US$500,000 - <US$3,000,000	8.03%	20
USD$3,000,000 or more	4.42%	11
TOTAL		249
